# Urinary Ascites Mimicking Portal Hypertension in a Healthy Woman With Bladder Microperforation

**DOI:** 10.7759/cureus.103438

**Published:** 2026-02-11

**Authors:** Dania Hailat, Catherine Colonna, Hayley Fowler, Leonidas Walthall

**Affiliations:** 1 Department of Internal Medicine, Medical University of South Carolina, Charleston, USA

**Keywords:** acute kidney injury, ascitic creatinine, bladder diverticulum, bladder microperforation, bladder rupture, portal hypertension mimic, reverse peritoneal dialysis, serum ascites albumin gradient, urinary ascites, urodynamics

## Abstract

Urinary ascites is a rare cause of ascites that can present with a high serum-ascites albumin gradient (SAAG), mimicking portal hypertension. Here we present a case of a 42-year-old woman with a history of cesarean section and a total laparoscopic hysterectomy (both remote and uncomplicated) that presented to the ED with abdominal ascites and pseudo-acute kidney injury after developing sudden, severe suprapubic pain during urination. Workup initially suggested a portal hypertension etiology due to a high SAAG; however, liver function and cardiac workup were normal. Her serum creatinine normalized after placing a Foley catheter; however, creatinine rebounded upon catheter removal, and urinary ascites was then confirmed by an elevated ascitic creatinine level. In follow-up, cystoscopy revealed a posterior bladder diverticulum with scar, demonstrating the likely culprit lesion of the bladder rupture. Urodynamics showed delayed bladder sensation due to dysfunctional voiding habits. The patient was advised to time voids, keeping volumes below 400-600 cc, and was scheduled for a six-month follow-up for uroflowmetry and post-void residual (PVR) measurement. This case highlights the importance of not overlooking urinary ascites in patients with unexplained ascites and concurrent apparent AKI, even in the absence of obvious risk factors such as this patient.

## Introduction

The diagnosis of ascites is traditionally classified according to the serum-ascites albumin gradient (SAAG) criteria, with high gradients pointing toward non-peritoneal etiologies, such as portal hypertension, and low gradients pointing toward peritoneal etiologies [1]. However, there are certain rare conditions that may not fit into these two categories. The genitourinary tract involves both the peritoneum and retroperitoneum. Thus, a rupture of the urinary tract can be a unique cause of ascites. Urinary ascites often presents similarly to portal hypertension, with elevated SAAG and clinical abdominal swelling and pain, usually in the setting of trauma or recent procedures [2]. Spontaneous bladder rupture is rare, but has been reported in cases such as vaginal delivery, malignancy, radiation, infection, and urinary retention, most often secondary to developmental delays or psychiatric illnesses [3-5]. In a patient with ascites and normal liver enzymes, it is important to consider urinary ascites secondary to bladder microperforation in the diagnostic differential. In this report, we present a case of urinary ascites in an otherwise healthy young female in the absence of traditional risk factors.

## Case presentation

A 42-year-old healthy woman with a history of two prior cesarean sections (in 2011 and 2016), total laparoscopic hysterectomy and bilateral salpingectomy (2021), and adenocarcinoma in situ status post-conization (2012), presented to the emergency room with acute, severe suprapubic pain. The pain began suddenly during urination, rated 10/10, radiated to her lower back, and was associated with nausea and shaking. It briefly subsided after voiding but returned just as severely with her next attempt to urinate, prompting an emergency department visit.

In the ED, she was afebrile, but her white blood cell count was 19,000 cells/µL. She was subsequently started on piperacillin-tazobactam (Zosyn; New York City, NY: Pfizer) for empiric antibiotic coverage, as well as multiple doses of pain medication. Urinalysis was normal except for trace hematuria. Renal CT was negative for nephrolithiasis. CT abdomen showed a small amount of free fluid in the lower abdomen and pelvis, but no obstruction or free air. After two days of observation, she was discharged with a presumed diagnosis of a passed kidney stone or an intermittent bowel obstruction. She was given a course of antibiotics and antispasmodics and was scheduled for outpatient abdominal imaging follow-up.

A few days later, she presented to another ED due to abdominal pain keeping her up at night despite taking hydrocodone every 6 h, accompanied by a distended abdomen. She was hemodynamically stable and afebrile. Labs showed hyponatremia (131 mEq/L), hyperkalemia (5.2 mEq/L), anion gap metabolic acidosis, and acute kidney injury with a creatinine of 3.5 mg/dL (baseline ~0.8 mg/dL). A transvaginal ultrasound showed a large volume of ascites, and renal US showed a distended left renal pelvis and a hyperechoic liver lesion. Diagnostic paracentesis showed ascitic fluid without evidence for spontaneous bacterial peritonitis. The SAAG was 3 g/dL, which raised concern for sinusoidal obstruction, such as cirrhosis, heart failure, or Budd-Chiari syndrome, rather than a low SAAG cause, such as peritoneal carcinomatosis or infection (Table 1). However, liver function tests and imaging were normal. BNP was undetectable, echocardiography was unremarkable, and hepatic vasculature was patent on Doppler. Low SAAG etiologies were also explored. However, TVUS was consistent with functional ovarian cysts, and CA-125 was negative. There was also no protein in the urine, making nephrotic syndrome unlikely as well. The etiology of ascites remained unclear.

**Table 1:** Ascitic fluid and serum analysis. Ascitic and paired serum studies showed a high SAAG of 3.0 g/dL in the absence of portal hypertension, and an elevated ascites-to-serum creatinine ratio of 3.43 during the evaluation of unexplained ascites. SAAG: serum-ascites albumin gradient; LDH: lactate dehydrogenase; PMN: polymorphonuclear neutrophils

Parameters	Ascitic fluid	Serum
Collection date	2/3/25	2/3/25
Appearance	Light yellow, clear	-
Volume removed (mL)	600	-
Albumin (g/dL)	0.6	3.6
Total protein (g/dL)	0.8	-
Glucose (mg/dL)	Not provided	-
LDH (U/L)	37	-
Creatinine (mg/dL)	12	3.5
SAAG (g/dL)	3 (3.6-0.6)	-
Ascites: serum creatinine ratio	3.43 (12/3.5)	
Total nucleated cells (cells/μL)	227	-
PMN count (cells/μL)	18	-
WBC count (cells/μL)	20	-
RBC count (cells/μL)	10	-
Differential	Neutrophils 18 cells/μL	-
	Lymphocytes 1%	-
	Macrophages 89%	-

**Figure 1:**
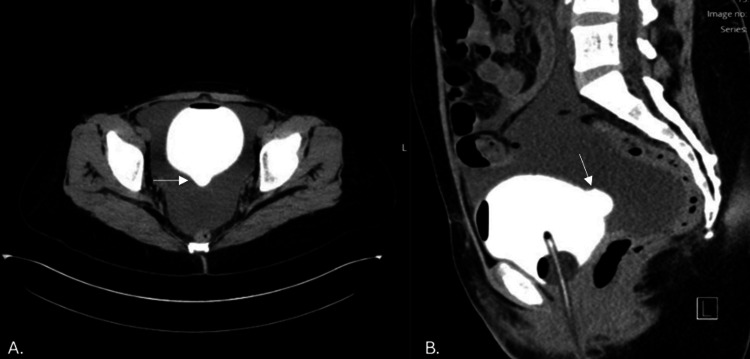
Cystogram: posterior bladder wall diverticulum identified by white arrows on cystogram (A) Frontal view showing an outpouching along the posterior bladder wall and (B) lateral view confirming the presence of a posterior bladder wall diverticulum.

**Figure 2:**
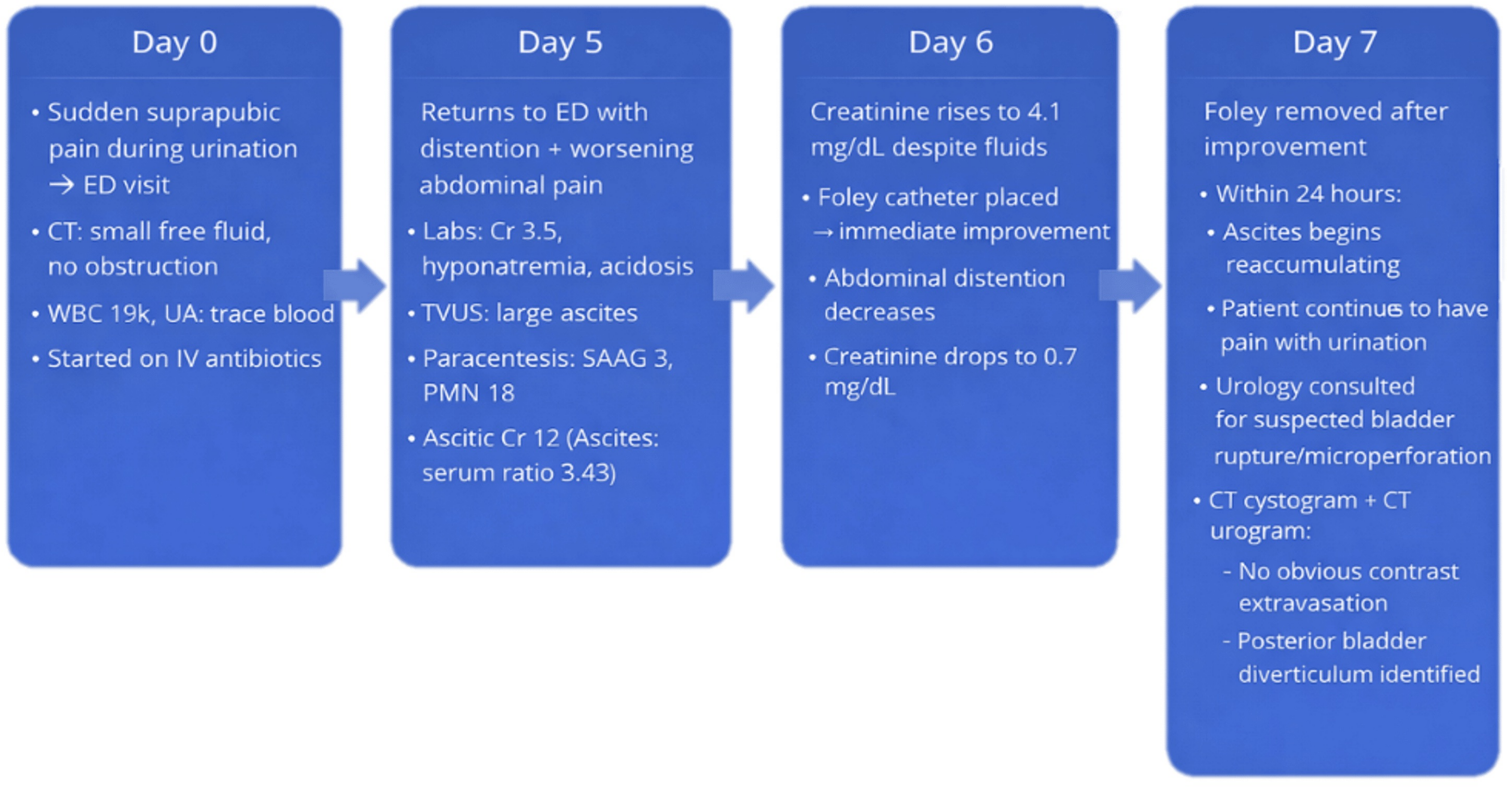
Timeline of the diagnostic course of urinary ascites. Timeline of the patient’s clinical course, from initial presentation with suprapubic pain and paracentesis findings, to abdominal distention following Foley catheter placement, to recurrence of ascites and creatinine elevation after catheter removal, raising suspicion for bladder microperforation, followed by improvement with catheter replacement, and ultimately identification of a posterior bladder diverticulum. The sequence highlights the diagnostic significance of paracentesis findings. UA: urinalysis; TVUS: transvaginal ultrasound; SAAG: serum-ascites albumin gradient; PMN: polymorphonuclear neutrophils; Cr: creatinine

Over the next two days, her creatinine rose from 3.5 mg/dL at admission to 4.1 mg/dL. After 600 mL paracentesis, IV fluids, and Foley catheter placement, her creatinine returned to 0.7 mg/dL. After Foley removal, her creatinine increased back to 1.4 mg/dL. Due to a rise in creatinine and fluid accumulating again after Foley removal in the context of pain while urinating, urology was consulted for a suspected bladder rupture. CT cystogram and CT urogram showed no obvious extravasation; however, a posterior bladder diverticulum was noted (Figures 1A, 1B). The patient also reported typical urinary habits and possible voiding dysfunction. This raised suspicion for bladder microperforation, and she was discharged with a bladder diary and outpatient urology clinic follow-up, as well as primary care physician (PCP), nephrology, oncology, and OB/GYN follow-ups (Figure 2).

At her urology appointment a few weeks later, she reported needing to void every 2-3 h due to abdominal discomfort, not urge, as well as right lower quadrant cramping in the first void of the day. Her post-void residual was low (5 mL), and her urinalysis was unremarkable. She reported a fluid intake of 4-5.5 L a day, with output of similar values. She was advised to reduce this to 2-2.5 L a day and was scheduled for follow-up.

Weeks later, she presented with an episode of gross hematuria with flank pain, nausea, fever, and suprapubic pain. Symptoms resolved with empiric antibiotics. Cystoscopy was performed and showed a posterior wall diverticulum with a stellate scar, suspicious for a possible prior site of bladder perforation in the setting of the patient’s two prior c-sections, a laparoscopic supracervical hysterectomy, and the patient's self-reported holding habits.

The following month, video urodynamics was performed, revealing a greatly delayed bladder sensation with the first sensation to void at 818 cc. She felt pain but not fullness at 1200 cc. She emptied completely and had no vesicoureteral reflux. In retrospect, the patient recalled that on the day the symptoms began, she had postponed urination until the afternoon, suggesting bladder overdistension, which could have contributed to a bladder microperforation in the setting of a previously injured bladder. She was advised to void every 2-3 h, maintain voided volumes of 400-600 cc, and return for follow-up in six months to assess uroflow and post-void residual (PVR).

## Discussion

New-onset ascites can be a clinical manifestation of various pathologies, many of which are not directly related to the liver; thus, it is important to adopt a systematic diagnostic approach to accurately ascertain the etiology. Urinary ascites is a rare phenomenon; therefore, a high index of suspicion is necessary to properly diagnose and timely treat patients with this condition [6]. In this case, the patient was a relatively young, otherwise healthy female who had a rapid onset of ascites and no obvious cause in the setting of concurrent elevated serum creatinine.

As with any new ascites presentation, the cornerstone of diagnostic evaluation is diagnostic paracentesis [7]. Classic fluid studies important for assessment during this procedure include cytology and cell count, albumin, total protein, and bacterial culture. While all of these studies are valuable, ascites protein and albumin are key to the diagnostic approach, as they enable categorization of the ascitic fluid [7]. Using ascites and serum albumin, clinicians can calculate the serum-ascites albumin gradient (SAAG), which helps determine whether ascites is secondary to portal hypertension (SAAG >1.1 g/dL) or due to a separate etiology (SAAG <1.1 g/dL). Examples of conditions that can cause elevated portal pressures in the absence of liver disease include heart failure, Budd-Chiari syndrome, or constrictive pericarditis, signifying increased hydrostatic pressure rather than liver failure. Low SAAG is seen with normal portal pressures and has an extensive differential diagnosis, including malignancy, TB, pancreatitis, nephrotic syndrome, or urinary ascites. Furthermore, analyzing protein in ascites can help further differentiate each category, as low ascites protein is often seen in cirrhosis and nephrotic syndrome. The patient in this case had an elevated SAAG of 3 g/dL, concerning for portal hypertension. Interestingly, there have been case reports documenting similar findings of urinary ascites depicting an elevated SAAG, typically consistent with a portal hypertension picture [2].

Of the above-mentioned lab work to consider with a diagnostic paracentesis, one that may commonly be overlooked is the ascites creatinine level. Urinary ascites is often attributed to trauma, recent urogynecologic surgery, or spontaneous bladder perforation [6,8]. Spontaneous bladder perforation has been reported in malignancy, infection, vaginal delivery, radiation, and urinary retention [3-5]. However, urinalysis, serologic data, and extensive imaging for this patient showed no evidence of any of these etiologies. Additionally, her gynecologic surgeries were remote (the most recent four years prior to presentation). Further detailed history elucidated concern for behavioral urinary retention, though our patient had no risk factors, such as developmental delay. Given the rarity of the condition, it is important to maintain a high level of suspicion in patients whose clinical presentation is atypical or whose paracentesis laboratory results are incongruent with subsequent findings. The patient in this case had an elevated SAAG, but the remainder of the portal hypertension workup was unremarkable, leading the team to question other etiologies. Supporting evidence that our patient was experiencing urinary ascites includes her elevated ascites/serum creatinine ratio of 2:9, improvement in creatinine with paracentesis and Foley catheter placement, and re-accumulation of ascites upon removal of her catheter.

The reabsorption of creatinine from ascites into serum, a process known as reverse peritoneal dialysis, causes elevated creatinine levels on serum blood tests and can lead to false assumptions of decreased renal function [4,9]. Rapid normalization and serum creatinine with Foley catheterization and improvement in ascites fluid volumes are also supportive of a urinary ascites diagnosis as follows: the patient in this case experienced normalization of serum creatinine in just one day after a urinary catheter was placed. Following catheterization, evaluation of the urinary tract for leak or perforation is performed with cystogram and urogram. Consultation with urology is reasonable, as any leaks or perforations would likely require surgical repair. In our patient, no leaks or perforations were identified on imaging; however, there was a diverticulum on the posterior bladder wall. There have been case reports documenting ruptured bladder diverticula leading to urinary ascites and pseudo-renal failure, so it is possible that her diverticulum had a microperforation that then resealed; however, no source was ever visualized on imaging [10]. Although bladder rupture is a known complication of recent abdominal or pelvic procedures, our case highlights that behavioral urinary retention may be a subtle risk factor that should be a subject of detailed history, and the index of suspicion for urinary ascites should remain high even in the absence of other traditional risk factors.

## Conclusions

Urinary ascites is an important differential to consider in patients with unexplained ascites, an elevated SAAG, and azotemia. This is especially important in those with a history of pelvic surgeries and/or dysfunctional voiding habits. Early recognition through a thorough history and ascites assessment, with ascites-to-serum creatinine ratio >1, can enable prompt diagnosis and treatment.
